# Which factors are associated with COVID-19 infection incidence in care services for older people in Nordic countries? A cross-sectional survey

**DOI:** 10.1177/14034948221085398

**Published:** 2022-05-12

**Authors:** Auvo S. Rauhala, Lisbeth M. Fagerström, Andrej C. Lindholst, Timo S. Sinervo, Tilde M. Bertelsen, Trond Bliksvær, Bente V. Lunde, Rolf Solli, Maria G. Wolmesjö, Morten B. Hansen

**Affiliations:** 1 Åbo Akademi University, Turku, Finland; 2 Vaasa Central Hospital, Finland; 3 University South-Eastern Norway, Norway; 4 Aalborg University, Aalborg East, Denmark; 5 Finnish Institute for Health and Welfare, Finland; 6 Nordland Research Institute, Bodo, Norway; 7 Nord University, Bodø, Norway; 8 University of Borås, Boras, Sweden

**Keywords:** COVID-19, COVID-19 testing, cross-sectional studies, Home Care Services, infection control, multilevel analysis, nursing homes, primary prevention, pandemics, Scandinavian and Nordic countries

## Abstract

**Aims:**

To investigate the differences between Sweden, Denmark, Finland and Norway regarding residential/home care units’ and frontline managers’ background factors, the resources allocated and measures taken during the initial phases of the COVID-19 pandemic, and whether and how these differences were associated with COVID-19 among older people in residential/home units.

**Methods:**

Register- and survey-based data. Responses from managers in municipal and private residential/home units. Number of municipal COVID-19 cases from national registries. Multilevel logistic multivariate regression analysis with presence of COVID-19 among older people in residential/home units as the outcome variable.

**Results:**

The proportions of residential/home units with client COVID-19 cases, mid-March–April 2020 were Denmark 22.7%, Finland 9.0%, Norway 9.7% and Sweden 38.8%, most cases found in clusters. The proportions were similar among employees. Client likelihood of having COVID-19 was six-fold higher if the employees had COVID-19. Mean client cases per residential/home unit were Denmark 0.78, Finland 0.46, Norway 0.22 and Sweden 1.23. For the same municipal infection incidence class, Sweden’s mean client infection levels were three-fold those of other countries. The regression analysis variables country, municipal COVID-19 incidence proportion, and care type were associated with client cases at *p* ⩽ .001. Compared with Denmark, the odds ratios (ORs) for Sweden, Norway and Finland were 1.86, 0.41 and 0.35 respectively. The variable difficulties in preventive testing had an OR of 1.56, *p* ⩽ .05.

**Conclusions:**

**Municipal COVID-19 incidence, employee cases, and the lack of testing resources somewhat explained the confirmed COVID-19 cases among older people in residential/home units. A two- to five-fold unexplained inter-country difference in ORs in the multivariate analyses was notable. The level of protection of vulnerable older clients in municipal and private residential/home units differed between the included countries.**

## Introduction

When seeking to minimize the health challenges associated with the COVID-19 pandemic, it has been critical to focus on protecting the most vulnerable groups. Older people in general and those in care are at higher risk of severe illness and mortality from COVID-19. While age has been exponentially associated with COVID-19 mortality, over one-third of such risk is linked to comorbidities or reduced muscle strength, measured by handgrip test [1, 2]. Overall, those aged ⩾75 have a 13-fold higher mortality risk from COVID-19 than those aged ⩽65 [1]. Older people in residential care settings appear to be highly susceptible to developing severe COVID-19 [2]. Fragile older people with multiple diseases in care constitute a high-risk group and moreover interact with numerous care providers, which further increases their risk [3]. In a cohort study of 627 long-term care facilities in Ontario, Canada, researchers found that the incidence rate ratio for COVID-19-related death for those aged ⩾69 was 13 times higher for care facility residents than community-living adults [4].

COVID-19 risk minimization in care services for older people appears to be linked to a variety of factors: administration, monitoring, information, staff and economic resources, continuity, infection prevention and control standards, testing and contact tracing, support for family, psychosocial well-being and continuous, effective governance [3]. We specifically examined two types of care facilities for older people: residential care settings (hereafter “residential units”) and home care for older people (hereafter “home units”).

The methods used to prevent COVID-19 infections have differed between the Nordic countries. Sweden implemented a relatively voluntary, delayed and less restrictive approach to preventive actions, while Denmark, Finland and Norway implemented stricter measures much earlier [5, 6]. Sweden has experienced the highest disease burden, both in terms of morbidity and mortality. The proportion of COVID-19 cases among people aged ⩾80 in Sweden has been about twice that of the other Nordic countries. In April 2020, 23% of employees across 22 residential units in Stockholm, Sweden, were found to be seropositive for COVID-19 [7].

## Aims

We investigated the differences between the included Nordic countries regarding residential/home units’ and frontline managers’ background factors and the resources allocated and measures taken during the initial phases of the COVID-19 pandemic, alongside whether and how these differences were associated with COVID-19 among older people in residential/home units.

The research questions were: 1. What are the inter-country differences between background variables, infection control resources and the measures implemented to prevent COVID-19 or control its outbreak among older people in residential/home units? 2. Which factors are associated with lower COVID-19 incidence among older people in residential/home units?

## Methods

### Study population

The study relied on register- and unique survey-based data. Our target population included all municipal and private residential/home units in Denmark, Finland, Norway and Sweden.

## The survey

Qualitative, exploratory, semi-structured interviews with frontline managers working in municipal and private residential/home units in Denmark were performed May–June 2020 to identify frontline managers’ range of actions and experiences of the COVID-19 pandemic. From the interviews, a web-based survey (SurveyXact, Rambøll Management Consulting, Denmark) consisting of items deemed comparable between all four included countries was designed and piloted.

The survey included 325 items, for example, residential/home unit and respondent background variables; sub-areas related to preventive measures, outbreak mitigation and resources (Table I and Supplementary Table I). Invitations to participate were sent to 3884 frontline managers working in municipal and private residential/home units in Denmark, Finland, Norway and Sweden, found either through a municipal registry (Finland, Norway, Sweden) or directly (Denmark); three remin-ders followed. Recipients (except for Denmark) were asked to forward the invitation to pertinent individuals within the relevant residential/home units (or residential/home units/other care organizations if a specific recipient name was unavailable). Data for variables were derived from 1300 to 1962 responses (not all respondents answered all questions). For example, in Norway, with 3155 frontline managers working in municipal or private institutional care and 4229 in home-based services [8], 786 invitations were sent and 343 responses were received. A similar pattern for the other included countries was seen, except for Denmark. According-ly, the response rate was only calculated for Denmark, which was 58% (860/1474). Responses from each municipality varied from 1 to 38. Represented in the data are 93% (91/98) of the municipalities in Denmark, 39% (121/310) in Finland, 41% (147/356) in Norway and 52% (151/290) in Sweden.

**Table I.** Participant characteristics and adequacy of COVID-19 control resources, Phase 2 (March 16–April 30, 2020).

Variable	Denmark		Finland		Norway		Sweden		Client C-19	Municipal C-19
Background variables	*n*	%	*n*	%	*n*	%	*n*	%	Corr	Corr
Leader group***										
Responsible for employees	584	(68.5)	146	(46.8)	113	**(34.5)**	322	*(75.6)*	**–.06** ^*^	.03
Responsible for other foremen	238	(27.9)	99	(31.7)	170	*(51.8)*	79	**(18.5)**	**.07** ^**^	–.00
Other management function	19	(2.2)	52	(16.7)	34	(10.4)	12	(2.8)	–.04	–.07**
Other position	12	(1.4)	15	(4.8)	11	(3.4)	13	(3.1)	–.02	–.03
Total	853	(100.0)	312	(100)	328	(100)	426	(100)		
Sector***										
Public	737	(85.7)	190	**(61.7)**	268	(81.0)	380	*(89.2)*	.02	–.04
Private for-profit	27	(3.1)	52	*(16.9)*	2	**(0.6)**	12	(2.8)	–.01	–.03
Private nonprofit	70	(8.1)	32	(10.4)	6	(1.8)	6	(1.4)	–.01	**.08** ^**^
Other	26	(3.0)	34	(11.0)	55	(16.6)	28	(6.6)		–.03
Total	860	(100.0)	308	(100.0)	331	(100.0)	426	(100.0)		
Service type***										
Home care or home and residential care unit	367	(42.7)	112	(36.4)	162	(48.9)	206	(48.4)	–.04	.02
Residential care unit	467	*(54.3)*	162	(52.6)	114	**(34.4)**	192	(45.1)	.04	–.02
Other	26	(3.0)	34	(11.0)	55	(16.6)	28	(6.6)		–.03
Total	860	(100.0)	308	(100.0)	331	(100.0)	426	(100.0)		
Previous experience of prevention and infection management**										
Yes as proportion of Total	681/842	(80.9)	222/289	(76.8)	238/281	(84.7)	300/402	(74.6)	**–.05** ^*^	.03
Experience from other healthcare sectors?***										
Yes as proportion of Total	551/837	(65.8)	226/289	*(78.2)*	178/280	(63.6)	207/396	**(52.3)**	–.02	–.00
**Educational level** (correlations as a continuous variable) ***									**.06** ^*^	**–.07** ^**^
Level 1	0	(0.0)	1	(0.4)	0	(0.0)	2	(0.5)		
Level 2	194	*(24.1)*	4	(1.4)	0	**(0.0)**	37	(9.5)		
Level 3	355	(44.1)	82	**(29.6)**	83	(29.7)	211	*(54.2)*		
Level 4	206	(25.6)	48	(17.3)	122	*(43.7)*	34	**(8.7)**		
Level 5	50	**(6.2)**	142	*(51.3)*	74	(26.5)	105	(27.0)		
Total	805	(100.0)	277	(100.0)	279	(100.0)	389	(100.0)		
Educational background in healthcare***										
Yes as proportion of Total	773/800	(96.6)	232/268	(86.6)	267/278	(96.0)	337/359	(93.9)	**–.11** ^**^	.04
**No. of employees frontline manager is responsible for, *n*, median**										
All respondents***	848	55	293	35	304	*98*	416	**34.5**	**.49** ^**^	–.02
Home care	362	52	107	45	157	*98*	203	**30**		
Residential care facility	466	60	160	**31**	112	*97*	189	37		
Only manages employees	608	50	157	**30**	125	*60*	325	35		
Lack of personal protective equipment as major problem**										
	384/687	(55.9)	141/223	(63.2)	145/226	(64.2)	139/278	(50.0)	.05	**.08** ^**^
Client access to systematic preventive COVID-19 testing as major problem***										
Yes as proportion of Total	222/653	*(34.0)*	40/221	(18.1)	31/216	**(14.4)**	78/262	(29.8)	.012**	**.15** ^**^

*Note*: Client C-19: no. of COVID-19 cases per residential/home unit; Municipal C-19: municipal COVID-19 incidence proportion (per 10,000). In variables with a range (%) between countries >15%: **(%)** = minimum, *(%)* = maximum; Corr: Pearson’s correlation coefficient. Municipal incidence proportion data taken from national registries, all other data from survey. Inter-country differences tested with chi-squared except number of employees, for which the Kruskal–Wallis test was employed.

* *p* ⩽ .05, **p ⩽ .01, ***p ⩽ .001, in bold.

## Data

Three separate phases between January and August 2020 were delineated. We present the survey data from Phase 2 (closure and reopening: mid-March–April 2020) because infections were most frequent during that period. Supplementary register-based data obtained from national statistical bureaus and authorities on daily (Denmark, Norway) or weekly (Finland, Sweden) number of municipal COVID-19 infections were also included. Data for municipal populations were also obtained.

## Variables

Most background variables were included in the statistical analyses in their original form. By combining service sector and service type questions, separate common categorical variables were created for both service sector and -type. Those units offering both home and residential care services were coded as home care. The numerous education variables were combined into an ordinal scale variable with five classes. A dichotomous education variable was also constructed to indicate whether a respondent’s educational background was in healthcare or not.

The register data from Norway on municipal COVID-19 incidence did not start until March 26, 2020, thus the infection numbers in Norway during Phase 2 were extrapolated using the coefficient 1.20, based on national statistics [9]. In Finland, a cumulative number of cases was reported only if five or more COVID-19 cases were registered. In Sweden, COVID-19 cases per municipality were reported only if the cumulative number was either zero or at least 15. Based on the cumulative number of cases in municipalities at the end of August, all zeros in Finland’s data were coded as “0”, while in Sweden’s data “<15” (but more than zero) for one phase was coded “7”, for two phases “3” and “11” and for all three phases “3”, “7” and “11”. A new incidence proportion was produced: municipal COVID-19 cases (per 10,000 inhabitants) for all three phases, including a categorical variable of this, with cut-off levels at 0, 3.5, 7.5, 15 and 30, based on percentiles.

## Statistics

Statistical analysis was conducted with IBM SPSS 25.00. *P*-values ⩽.05 were considered statistically significant. In descriptive analyses, categorical variables were presented with absolute and relative frequencies (percentage), and continuous variables with mean or median. Missing data were not addressed. Inter-country differences were tested, without pairwise comparisons, using chi-squared, except for the number of employees, which was tested using the Kruskal–Wallis test. Bivariate correlations were estimated with Pearson’s method.

Multivariate modeling was used to explain the dichotomous outcome variable: the presence of COVID-19 among older people in residential/home units. Due to the hierarchal structure of the data, multilevel modeling was applied using the generalized mixed linear models procedure, where a binomial logit model was employed for binary multilevel logistic regression [10]. Random effect was included as a random intercept, and municipality was treated as a Level 2 variable. All other independent variables were entered as fixed factors. Variance components was used as the covariance structure. Country was not treated as a Level 3 variable because there were too few countries included in the analysis [11]. Odds ratios (ORs) and their 95% confidence intervals (CIs) were estimated for predictors. *P*-values were presented with levels ⩽.05, ⩽.01 and ⩽.001. The properties of the overall model were reported with model *p*, correct classification (%) and the Akaike corrected information criterion (AICc). Collinearity was tested. All two-way interactions of the models’ variables were analyzed. Country was included in all models. Other variables were removed in the model one section at a time. All preventive and mitigating measure variables were tested separately with country variable. Municipal COVID-19 incidence proportion (per 10,000) was centered on the grand mean (11.868), to make the main effect of country more interpretable. Data on the number of older people receiving care in municipal and private residential/home units (hereafter “client(s)”) was unavailable, thus we had no data on the exact client incidence proportion of COVID-19. Therefore, instead of the number of clients per unit, part of the analyses was controlled for a proxy variable: the number of employees a frontline manager was responsible for.

All independent variables and interaction terms that had shown statistical significance in previous models were entered into the final model. Thereafter, backward stepwise selection was employed until only variables and interactions with a significant association were left.

## Ethics

Answering the survey was voluntary. Other than what is listed in Table I and the email addresses for those respondents who requested the survey be sent directly to them, no personal information was collected. By participating in the survey, respondents consented to the use of their data in the research material. Respondents were informed in the invitation that the data would be processed and reported in such a way that individual respondents could not be identified.

## Results

### Descriptive statistics of background, resource and measure variables

Descriptive statistics of the background variables are presented in Table I. Ninety-two percent (1810/1962) of respondents were female. Over four-fifths of the included residential/home units were public sector settings. Frontline managers’ most common role was having responsibility for their employees. Most frontline managers had an educational background in healthcare and experience of healthcare and infections. Educational level varied remarkably between countries. Level 5 (Master’s degree) was much more common in Finland than in other countries. Frontline managers were responsible for a median of 35–55 employees, except in Norway where the median was twice this.

Descriptive statistics of infection control resources are also presented in Table I. Most respondents considered a lack of personal protective equipment to be a major problem. About a fifth considered access to COVID-19 testing to be a major problem, with the highest proportions seen in countries also recording the highest COVID-19 incidence.

Measures to prevent or mitigate the outbreak of COVID-19 are presented in Supplementary Table I. Overall, two-thirds of preventive measures were fairly uniform across all included countries. Similar data were seen on mitigating measures. Most inter-country differences were statistically significant.

### Descriptive statistics of COVID-19 incidence in residential/home units

Confirmed COVID-19 among clients in residential/home units was 22.7% (159/699) of units in Denmark, 9.0% (21/234) in Finland, 9.7% (23/237) in Norway, 38.8% (100/258) in Sweden and 21.2% in total. The proportions among employees were mostly at the same level or slightly higher than those of clients. There was a significant association (*p* < .001) between infections in employees and clients. If employees had COVID-19, in 62% of units at least one client also had COVID-19. Conversely, if employees did not have COVID-19, clients had COVID-19 in only 10% of units.

**Figure 1.**
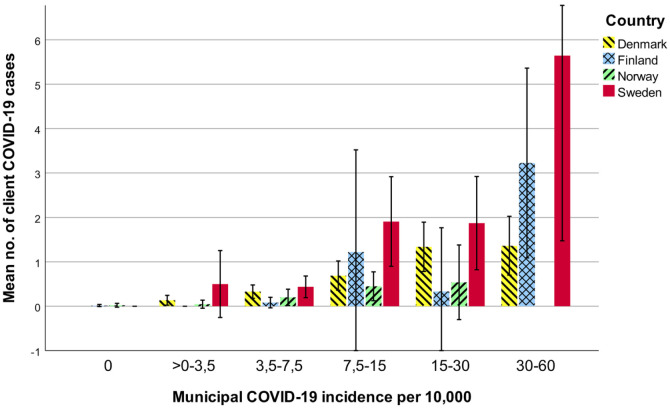
Mean number of client COVID-19 cases per unit and their 95% confidence intervals in municipal incidence proportion (per 10,000) classes presented by country, Phase 2 (March 16–April 30, 2020). Some parts of confidence intervals lie outside Figure. Municipal incidence data are from national registers, all other data from survey. Only groups with *n* ⩾ 9 are presented.

The mean number of client cases per residential/home unit was 0.78 in Denmark, 0.46 in Finland, 0.22 in Norway, 1.23 in Sweden and 0.91 in total. These numbers are presented in classes according to confirmed municipal COVID-19 incidence proportion (Figure 1). Excepting class zero, for these classes Sweden’s mean number of client cases was on average 3.1 times higher than other countries’ means. More than one COVID-19 cases were found in 68% of residential/home units with clients with COVID-19 and in 28.1% of those with at least five clients with COVID-19. Client cases increased as the incidence in a municipality increased. The mean confirmed municipal COVID-19 incidence proportion (cases per 10,000 inhabitants) was 13.3 in Denmark, 5.1 in Finland, 6.4 in Norway and 17.8 in Sweden.

### Bivariate correlations

Several background, resource and measure variables correlated with the included residential/home units’ client COVID-19 cases (Table I and Supplementary Table 1), but the coefficients were small and not in a consistently preventive direction. Additional correlation analyses were performed because of the unexpected positive correlation of educational level with residential/home unit client COVID-19 incidence (*r* = .06). Both variables had a low positive correlation with municipal population (*r *= .03 and *r *= .11, respectively) and a more marked correlation with the number of employees a frontline manager was responsible for: .19 and .49, respectively.

### Multilevel logistic regression models

In multilevel logistic regression analysis (Table II and Supplementary Table 2), adding municipal COVID-19 incidence proportion to the variable country (Model 2) improved model fit according to AICc. Of all the preventive measure variables tested with the variable country, only one variable (visit/exit restrictions) had ORs < 1 and *p* < .05 (Model 6). Several other variables with a low positive correlation (Supplementary Table 1) had quite high ORs, 2.35–4.27; *ps* were associated, .000–.002.

**Table II.** Final multilevel logistic regression model explaining client Covid-19, Phase 2 (March 16–April 30, 2020).

	**Model 7**
**Variables and parameters**	Final model
**Model parameters**	
*n*	1036
AICc	5123
Overall *p*	.000
Correct classification (%)	82.8
**Fixed effects**, OR, (CI), *p*	
**Country**	***
Sweden	**1.86 (1.17**–2.96)**
Norway	**0.41 (0.22**–0.78)**
Finland	**0.35 (0.16**–0.75)**
Denmark (ref.)	ref.
**Municipal C-19 incidence**	**1.06 (1.05**–1.08)***
**Service type**	***
Home care or home and residential care unit (ref.)	ref.
Residential care unit	**0.41 (0.29**–0.59)***
Other	0.35 (0.12–1.00)
**No. of employees frontline manager is responsible for (in 10s)**	**1.02 (1.01**–1.04)***
**Lack of preventive client testing**	**1.56 (1.07**–2.26)*

*Note*: Municipal incidence data are from national registries, all other data are from survey. Municipal C-19 incidence = municipal COVID-19 incidence proportion per 10,000; *AICc* = Akaike corrected information criterion; OR = odds ratio; CI = 95% confidence interval; municipality as random effect (intercept), that is, Level 2 variable; dichotomous variables: reference category = No.

ORs and their CIs with *p* ⩽ .05 in bold.

* *p *⩽ .05, **p ⩽ .01, ***p ⩽ .001.

In the final model (Model 7, Table II), only the variables of country, municipal COVID-19 incidence proportion and problems with the preventive COVID-19 testing of clients remained. The final model was controlled for unit type and number of employees a frontline manager is responsible for. The *p* for country effect was ⩽.001 and ORs were 1.86 (*p* ⩽ .01) for Sweden, 0.41 (*p* ⩽ .01) for Norway and 0.35 (*p* ⩽ .01) for Finland.

## Discussion

### Main findings

From the survey data, we saw that one-fifth of all residential/home units in the countries investigated had confirmed client COVID-19 cases during Phase 2, of which most appeared in clusters. If employees had COVID-19, clients’ COVID-19 likelihood was six times higher. Both municipal and client COVID-19 incidences were clearly highest in Sweden. However, the higher municipal incidence in Sweden seemed to only partially explain the higher client incidence seen there. Sweden’s client infection levels remained about three-fold those of the other countries with the same municipal infection incidence class.

Clear differences between the included countries for several background variables were seen, for example, frontline managers’ educational level and residential/home units’ size, sector and unit type. While respondents’ perception of a lack of personal protective equipment was equally common in all included countries, most of those who perceived the lack of preventive client COVID-19 testing to be a major problem were from countries with the highest confirmed COVID-19 incidence. About two-thirds of the preventive measures we examined were in use in all countries.

In all multivariate models, the country effect on client COVID-19 incidence was clearly seen; in the final model, Sweden’s OR was two- to five-fold higher than the other included countries. Municipal COVID-19 incidence and the lack of client preventive testing were other explanatory factors; service type and number of employees a manager was responsible for were controlled for.

### Previous studies

Other researchers have observed quite similar results to those presented in this study for nursing homes [12] and skilled nursing facilities [13]. The associated factors they have observed include number of beds, urban/rural, client ethnic background and state. They furthermore have reported on non-associated factors such as staffing, ownership and quality ratings. Low population density may account for the low COVID-19 incidence observed for Finland and Norway in this study.

Some researchers have stressed the significance of rapid, symptom-triggered testing and universal testing with proven outbreaks, because of the high proportion of asymptomatic COVID-19 cases [14-17]. Infection predictability on the single client or facility level can thus be construed to be poor, because of such asymptomatic cases and infection clusters. This is in line with our results and supports the need for adequate testing resources. Also consistent with our results is other researchers’ observations that the proportion of COVID-19 cases among people aged ⩾80 in Sweden has been about twice that of same age-group cases in other Nordic countries [6].

### Implications

Unpredictability in a pandemic situation requires effective and rapid infection control efforts such as mass testing or symptom screening, especially in units for vulnerable clients. This is particularly important in situations with high municipal incidence or the presence of COVID-19 infections among those who interact with older people. During the first wave of the ongoing pandemic, there was a major shortage of personal protective equipment and testing capacity in many countries [18]. Better preparation for future pandemics is needed. The client COVID-19 incidence revealed in this study was found to be only partially associated with the level of COVID-19 in the surrounding environment. The country context also appeared to be remarkably aligned with confirmed client COVID-19 cases in the settings explored in this cross-sectional study. Even after controlling for all statistically significant variables, the OR of incidence in Sweden was still seen to be about two- to five-fold higher than that of the other included Nordic countries. Further investigation is needed to clarify such country-level differences.

### Strengths and limitations

Our results were largely consistent with others’ results and considered to be largely generalizable to (at least) other Western countries with sufficiently similar health, social care, cultural and economic systems. Except for Denmark, it was not possible to calculate the exact response rate. Selection bias is possible. Furthermore, respondents perhaps did not provide or could not remember correct information; they also may have tired of answering the survey. We found, however, that the proportion of municipalities represented in our data, seen as 50% of municipalities across all included countries, was sufficient for our analyses.

Direct causal conclusions from the associations between variables should not be drawn, because of the study’s observational, cross-sectional design. Associations other than success in preventing client COVID-19 were found between variables: several variables were associated with municipal COVID-19 incidence, some with facility/unit/municipal size. The mean unit size for Norway was twice as large as the unit size for the other included countries, which had an impact on the univariate – but not multivariate – comparisons of data related to unit infection rates. Different national registration and test practices made inter-country comparison of confirmed COVID-19 cases problematic. Of the included countries, Denmark performed the most COVID-19 tests and Sweden the least [19].

We observed a positive correlation between most of the measured variables with reported client COVID-19 cases per unit. One explanation may be that unit infections or increased unit infection risk were associated with increased use of preventive measures.

## Conclusions

Using an observational, cross-sectional design to explain COVID-19 cases among older people in residential- or home units was not without problems. To some degree, the variables measured in this study – municipal COVID-19 incidence, employee cases, lack of testing resources – could be considered successful in this endeavor. Nonetheless, a two- to five-fold unexplained inter-country difference in ORs was notable. The level of protection of vulnerable older clients in municipal and private residential/home care units differed between the included Nordic countries. Country context is clearly important and alongside mortality should be further explored in future studies.

## Supplemental Material

sj-xls-1-sjp-10.1177_14034948221085398 – Supplemental material for Which factors are associated with COVID-19 infection incidence in care services for older people in Nordic countries? A cross-sectional survey Click here for additional data file. Supplemental material, sj-xls-1-sjp-10.1177_14034948221085398 for Which factors are associated with COVID-19 infection incidence in care services for older people in Nordic countries? A cross-sectional survey by Auvo S. Rauhala, Lisbeth M. Fagerström, Andrej C. Lindholst, Timo S. Sinervo, Tilde M. Bertelsen, Trond Bliksvær, Bente V. Lunde, Rolf Solli, Maria G. Wolmesjö and Morten B. Hansen in Scandinavian Journal of Public Health
